# An experimental investigation of association between children’s lying and behavior problems

**DOI:** 10.3389/fpsyg.2022.982012

**Published:** 2022-08-22

**Authors:** Xue Liu, Siyuan Shang, Sarah Zanette, Yongkang Zhang, Qingzhou Sun, Liyang Sai

**Affiliations:** ^1^Department of Psychology, School of Education, Hangzhou Normal University, Hangzhou, China; ^2^Mental Health Counseling Center, Zhejiang University of Science and Technology, Hangzhou, China; ^3^Luther College, University of Regina, Regina, SK, Canada; ^4^School of Management, Zhejiang University of Technology, Hangzhou, China

**Keywords:** lying, deception, children, behavior problem, moral

## Abstract

Children’s lying is a major concern for parents and teachers alike, not only because lying is an antisocial behavior but also because children’s lying correlates with other behavior problems, such as aggression and delinquency. Despite considerable correlational evidence demonstrating the relation between children’s lying and behavior problems, experimental evidence is scarce. This study uses a novel task to experimentally examine the relation between lying for personal reward and behavior problem symptoms among 9- to 11-year-old typically developed children (*N* = 275, 139 boys). Results revealed a positive correlation between children’s lying for personal reward and their behavior problem symptoms, and this correlation increases with age. Overall, this study provides experimental evidence suggesting children’s lying for personal reward is associated with behavior problems.

## Introduction

Children begin to lie as early as 2 years of age, and their frequency of lying increases throughout the preschool period (Lee, 2013). This developmental increase in lying is not a significant concern, as lying at this age is considered a milestone of normal cognitive development (Talwar and Lee, 2008; Evans and Lee, 2011; Ding et al., 2015). Indeed, considerable evidence has revealed that children’s lying correlates with crucial cognitive skills such as theory of mind and executive functioning (for a meta-analysis, see Sai et al., 2021).

With the development of moral awareness and self-control ability, children’s tendency to antisocial lie typically decreases as they move through middle childhood (Glätzle-Rützler and Lergetporer, 2015; Carl and Bussey, 2019; Sai et al., 2022). However, some children who do not follow this decreasing trajectory in middle childhood and instead continue to tell frequent lies at a high rate are considered as problematic (Talwar and Crossman, 2011). Indeed, a number of studies have revealed that children’s lying correlates with various behavior problems like aggression, fighting, and delinquency (Stouthamer-Loeber, 1986; Gervais et al., 1998; Engels et al., 2006; Ostrov, 2006; Zanette et al., 2020). However, the majority of these studies relied on reports from parents, teachers, or clinicians as a measure of the frequency of children’s lies. As highlighted by Mugno et al. (2019), the reports of observers like parents or teachers may be influenced by a variety of factors, such as their own feelings about or knowledge of a child’s overall behavior patterns, especially when the child displays symptoms of behavior problem. Moreover, due to the inherent nature of lying being deceptive, skilled lie-tellers might not always be caught. In fact, considerable research has shown that adults are notoriously poor detectors of children’s lies (for meta-analysis, see Gongola et al., 2017), making it even more likely that their estimates of children’s lying are skewed. It therefore remains unclear whether the correlation between children’s actual lying behaviors and their behavior problems truly exists.

One way to address the above-mentioned limitations is to use experimental paradigms to assess children’s lying and its relation to behavior problems. To the best of our knowledge, only few studies have explored the relation between children’s lying and their behavior problems using experimental paradigm.^1^ For example, Lavoie et al. (2018) used a paradigm in which children first received an incentive to cheat to win a prize and then the experimenter asked them whether they cheated to determine whether children would lie to conceal their cheating; their findings revealed that children with behavior problem symptoms were more likely to tell a lie to conceal their cheating.

Although Lavoie et al. (2018) suggested that children’s lying to conceal their cheating correlated with their behavior problems, it remains unclear whether other types of antisocial lies correlate with their behavior problems. Lying for personal reward is one of the antisocial lies that can damage society (Talwar and Crossman, 2011), and it is also a symptom that appears on several rating scales of childhood disorder, including Rutter Children Behavior Questionnaire, Child Behavior Checklist, and Strengths and Difficulties Questionnaire (Rutter, 1967; Goodman and Scott, 1999; Mugno et al., 2019). Nevertheless, limited experimental studies explored the correlation between children’s lying for personal reward and behavior problems.

This study aims to use an experimental paradigm to investigate lying for personal reward and its relation with behavior problems among a set of typically developed children. Moreover, we also want to examine whether the correlation between lying and behavior problems varies with age. To date, only one study has examined this question and they found that the relation between children’s lying and their behavior problems increases with age (Stouthamer-Loeber and Loeber, 1986). However, children’s lying was measured by reports from parent and teachers in that study, it remains unclear whether this result still holds when an experimental paradigm is used to measure children’s lying.

To assess children’s spontaneous lying for personal reward, a *number-guessing game*, which was modified from behavioral economic research, was used (Kajackaite and Gneezy, 2017). In the task, children think of a number from 1 to 6 and then roll a die and report whether their secret number matches what the die is showing. Participants are rewarded for each match they report, giving them an incentive to lie. An advantage of this paradigm is that it enables assessing children’s spontaneous lying without the fear of being detected. Based on the previous literature (Stouthamer-Loeber, 1986; Lavoie et al., 2018), we expected that children’s lying for personal gain would be positively related to their behavior problem and would be more closely related to behavior problem with age.

## Materials and methods

### Participants

A total of 275 school-aged children (*M*_*age*_ = 9.31 years; SD = 1.53, range = 6.57-11.96) were enrolled in this study, including 139 boys (*M*_*age*_ = 9.35 years, *SD* = 1.48, range, 6.57–11.96) and 136 girls (*M*_*age*_ = 9.26 years, *SD* = 1.57, range = 6.64–11.91). This sample size was based on a prior power analysis using G*Power 3.1 with Power (1 – β) set at 0.95 and α = 0.05 (Faul et al., 2009). The results revealed that to detect an *R*^2^ increase in multilevel regression with a medium effect size (*f*^2^ = 0.05), 262 participants would be needed. This effect size (*f*^2^ = 0.05) was chosen based on the effect size from Lavoie et al. (2018).

### Procedure

All participants were tested individually in a quiet room at their school. Children played a number-guessing game with an experimenter. Teachers were asked to fill the Rutter Children Behavior Questionnaire (RCBQ) to assess children’s behavior problems.

### Number-guessing game

To examine children’s lying behavior, we used a number-guessing game that was based on the *mind game* used by Kajackaite and Gneezy (2017). Participants were informed that they could win tokens for correctly guessing the number on a die and that any tokens they won could be exchanged for prizes (i.e., a pen) at the end of the session. In each of the six trials, they were asked to think of a secret number between 1 and 6 and remember it. Then, they rolled a standard six-sided die and reported whether their secret number matched what the die showed. Each time they reported “same,” they won a token. If children reported truthfully, the probability of rolling the secret number in a single trial is 1/6 (see also Weisel and Shalvi, 2015). The expected number of trials for which children reported “same” in six trials is 1, and lying was assessed by comparing the number of trials for which children reported “same” to the expected number.

### Children’s behavior problems

To examine children’s behavior problem, the teacher-reported RCBQ was used (Rutter, 1967); this questionnaire was usually used for children aged 7–13 years and it comprises 26 items that capture children’s behavior problems at school, including both antisocial behavior (e.g., the behavior of destroying oneself and others, or not being disciplined) and neurotic behavior (e.g., problem behaviors like being upset or refusing to go to school). The teachers were asked to rate, on a 3-point scale, ranging from 0 (*does not apply*) to 2 (*certainly applies*), how well each item described children in their class. The internal consistency was α = 0.60. We used the sum score of the children’s behavior problem.

## Results

### Lying for personal reward

Children reported 2.58 “same” on average, 57.5% of them reported 3 or more “same”, and a one-sample *t*-test revealed that children reported more “same” than expected (*t*_(274)_ = 18.39, *p* < 0.001), suggesting that children falsely reported their outcome. Furthermore, Pearson correlation analyses revealed a significantly negative correlation between age and the number of trials in which children reported “same,” suggesting that as age increased children were significantly less likely to have lied (*r* = –0.56, *p* < 0.001).

### Children’s lying and behavior problems

For all children attended the study, their scores on behavior problems ranged from 0 to 13, *M* = 2.28, *SD* = 2.49. To examine the relation between children’s lying and behavior problems, a hierarchical regression was performed with behavior problems as the predicted variable. While gender was entered in the first step as a control variable, children’s age and lying score were entered in the second step, and the interaction between age and lying score was entered in the third step. The results revealed that the first model was not significant [*F*_(1_,_273)_ = 0.77, *p* = 0.382, *R*^2^_*adj*_ = 0.03 (model 1); Table 1], but the second model was significant [*F*_(3_,_271)_ = 12.38, *p* < 0.001, *R*^2^_adj_ = 0.11 (model 2); Table 1], in which both children’s age (β = 0.24, *t* = 3.51, *p* = 0.001) and lying (β = 0.42, *t* = 6.02, *p* < 0.001) positively correlated with their behavior problems.

**TABLE 1:** Hierarchical regression analyses for predictors of children’s behavior problems.

Model		β	*t*	*P*	Δ*R*^2^	Δ*F*	*p*
Model 1	Gender	–0.05	–0.88	0.382	0.003	0.77	0.382
Model 2	Age	0.24	3.51	0.001	0.12	18.13	< 0.001
	Lying	0.42	6.02	< 0.001			
Model 3	Interaction	0.19	3.19	0.002	0.03	10.15	0.002

The third model was also significant [*F*_(4_,_270)_ = 12.13, *p* < 0.001, *R*^2^_adj_ = 0.14 (model 3)], and a significant change was noted between models 2 and 3 (Δ*R*^2^ = 0.03, Δ*F* = 10.15, *p* = 0.002). Specifically, the interaction between age and lying score was significant (β = 0.019, *t* = 3.19, *p* = 0.002), suggesting that the correlation between children’s lying and their behavior problems differ as a function of age.

**FIGURE 1:**
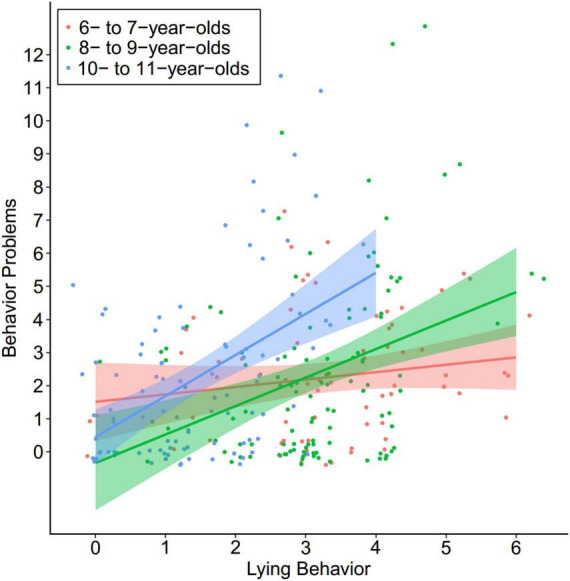
Relation between children’s lying and their behavior problems (jitter plot with fitted line and 95% CI).

To further investigate the impact of age on the correlation between lying and behavior problems, children were split into three age groups (6- to 7-year-olds; 8- to 9-year-olds; and 10- to 11-year-olds). We performed hierarchical regressions in each age group with behavior problems as the predicted variable, gender as a control variable in the first step, and lying scores as an independent variable in the second step. The results revealed that (Table 2), the correlation between lying and behavior problems was not significant among 6- to 7-year-olds (β = 0.17, *t* = 1.32, *p* = 0.192), but significant in both 8- to 9-year-olds (β = 0.35, *t* = 3.89, *p* < 0.001) and 10- to 11-year-olds (β = 0.47, *t* = 5.11, *p* < 0.001; Figure 1).

**TABLE 2:** Hierarchical regression analyses for predictors of children’s behavior problems in each age group.

Age Group	Model		β	*t*	*P*	Δ*R*^2^	Δ*F*	*p*
6- to 7-year-olds	Model 1	gender	–0.05	–0.40	0.692	0.002	0.16	0.692
	Model 2	lying	0.17	1.32	0.192	0.027	1.74	0.192
8- to 9-year-olds	Model 1	gender	-0.04	–0.43	0.667	0.002	0.19	0.667
	Model 2	lying	0.35	3.89	< 0.001	0.121	15.13	< 0.001
10- to 11-year-olds	Model 1	gender	–0.07	–0.67	0.505	0.005	0.45	0.505
	Model 2	lying	0.47	5.11	< 0.001	0.218	26.11	< 0.001

## Discussion

This study used an experimental task to explore the relation between children’s lying and their behavior problems. As expected, we found that children’s lying for personal reward positively correlated with their behavior problems, and this correlation increases with age. These findings provided experimental evidence about the relation between children’s actual lying behavior and their behavior problems.

We found that children’s lying for personal reward positively correlated with their behavior problem. This result furthers previous self-reported studies and provides experimental evidence that children’s actual lying for personal reward correlates with behavior problems. Zanette et al. (2020) reported that children with more significant conduct problems believe that other people tell lies more often than children with fewer conduct problems. Thus, one likely reason for the correlation is that children with more significant behavior problems believe other people also lie and, thus, they also lie more themselves. Another likely reason is that children’s lying could reflect deficient development of cognitive abilities like self-control ability (Talwar and Crossman, 2011), and this poor self-control ability reportedly correlated with behavior problems (Fergusson et al., 2013; Rhee et al., 2016, 2018). Future studies should continue examining these hypotheses. Lavoie et al. (2018) used an experimental paradigm to examine children’s lying to conceal transgression and reported that children with behavior problems told more lies to conceal their transgression. All these findings indicate that both lying for personal reward and lying to conceal their transgression correlate with their behavior problems. Furthermore, we found that the correlation between lying for personal reward and behavior problems increases with age. This result is consistent with one previous finding (Stouthamer-Loeber, 1986). One likely explanation is that children tend to lie less with age because of development of moral consciousness and self-control ability, while older children who still tell frequent lies might not develop those abilities well and thus, have more behavior problems (Talwar and Crossman, 2011). It should be noted that few studies have examined the moderating effect of age on the link between children’s lying and behavior problems, and more studies are needed to test this question in the future.

This study has several limitations worth acknowledging. First, the task we used in this study was not allowed to evaluate children’s lying in a specific trial; thus, further studies should create new tasks to address this issue. Second, it is still unclear the causal correlation between children’s lying and their behavior problems, further studies could use longitudinal designs to examine the causal correlation between children’s lying and behavior problems. Third, the internal consistency of teacher-reported RCBQ in this study was only 0.6, future studies may consider using other behavior problems scales such as children behavior checklist (CBCL) to test this question again (e.g., Zanette et al., 2020). Four, previous studies have shown that normal developing children’s lying is correlated with their cognitive abilities such as executive function and theory of mind (e.g., Fu et al., 2018; Sai et al., 2021). Further studies should explore whether such factors influence the lying of children with behavior problems. Finally, behavior problems involve various types of problem behaviors such as aggression or delinquency, further studies are needed to experimentally examine the correlation between lying and those specific problem behaviors.

Overall, this study uses an experimental paradigm to explore children’s lying for personal reward and its correlation with behavior problems. The findings establish that children’s lying for personal reward correlates with their behavior problem symptoms, and this correlation increases with age. Hence, this study provides experimental evidence about the correlation between children’s actual lying and behavior problems.

## Data availability statement

The datasets presented in this study can be found in online repositories. The names of the repository/repositories and accession number(s) can be found below: https://osf.io/dphfq/?view_only=6ef94928c6a84f2582e70e5fc2338aa7.

## Ethics statement

The studies involving human participants were reviewed and approved by Human Research Ethics Committee of the Hangzhou Normal University (Ethics approval number: 2019-011). Written informed consent to participate in this study was provided by the participants’ legal guardian/next of kin.

## Author contributions

XL and SS collected and analyzed the data, and drafted the manuscript. SZ wrote the manuscript. YZ collected the data. QS and LS conceived the idea and wrote the manuscript. All authors contributed to the article and approved the submitted version.

## Footnotes

1 Notably, Mugno et al. (2019) used experimental paradigms to investigate both lying to conceal transgression and lying for personal reward among children with a disruptive disorder. Lavoie et al. (2018) and this study examined typical children with behavior problems symptoms.
